# GrTEdb: the first web-based database of transposable elements in cotton (*Gossypium raimondii)*

**DOI:** 10.1093/database/bax013

**Published:** 2017-03-24

**Authors:** Zhenzhen Xu, Jing Liu, Wanchao Ni, Zhen Peng, Yue Guo, Wuwei Ye, Fang Huang, Xianggui Zhang, Peng Xu, Qi Guo, Xinlian Shen, Jianchang Du

**Affiliations:** 1 Key Laboratory of Cotton and Rapeseed (Nanjing), The Institute of Industrial Crops, Jiangsu Academy of Agricultural Sciences, Nanjing 210014, China; 2 Provincial Key Laboratory of Agrobiology, The Institute of Biotechnology, Jiangsu Academy of Agricultural Sciences, Nanjing 210014, China; 3 State Key Laboratory of Cotton Biology, The Institute of Cotton Research, Chinese Academy of Agricultural Sciences, Anyang 455000, China

## Abstract

Although several diploid and tetroploid *Gossypium* species genomes have been sequenced, the well annotated web-based transposable elements (TEs) database is lacking. To better understand the roles of TEs in structural, functional and evolutionary dynamics of the cotton genome, a comprehensive, specific, and user-friendly web-based database, *Gossypium raimondii* transposable elements database (GrTEdb), was constructed. A total of 14 332 TEs were structurally annotated and clearly categorized in *G. raimondii* genome, and these elements have been classified into seven distinct superfamilies based on the order of protein-coding domains, structures and/or sequence similarity, including 2929 *Copia-like* elements, 10 368 *Gypsy-like* elements, 299 *L1*, 12 *Mutators*, 435 *PIF-Harbingers*, 275 *CACTAs* and 14 *Helitrons*. Meanwhile, the web-based sequence browsing, searching, downloading and blast tool were implemented to help users easily and effectively to annotate the TEs or TE fragments in genomic sequences from *G. raimondii* and other closely related *Gossypium* species. GrTEdb provides resources and information related with TEs in *G. raimondii*, and will facilitate gene and genome analyses within or across *Gossypium* species, evaluating the impact of TEs on their host genomes, and investigating the potential interaction between TEs and protein-coding genes in *Gossypium* species.

**Database URL:** http://www.grtedb.org/

## Introduction

Transposable elements (TEs) are the most abundant DNA components in most characterized genomes of high eukaryotes (1). Based on their structural features and transposition mechanisms, TEs are generally classified into two classes: retrotransposons and DNA transposons (2). In plants, retrotransposons are further classified into two distinct orders, long terminal repeat (LTR)-retrotransposons (*Ty1/Copi*a and *Ty3/Gypsy*) and non-LTR retrotransposons (*LINE* and *SINE*), whereas DNA transposons are traditionally separated into two main orders, terminal inverted repeat (TIR) (*Tc1-Mariner*, *hAT*, *Mutator*, *PIF/Harbinger* and *CACTA*) and Helitron (*Helitron*) (2, 3). Although TEs are often considered as ‘junk DNA’ due to their continuous reproduction and potential disruption of the regular host genes (4–6), more evidence has unambiguously shown that they play important roles in altering gene structures, regulation of gene expression, affecting genome evolution and creating new genes (7–9). Thus, complete identification and characterization of TEs have become a priority in genome sequencing projects, and this will largely contribute to accurate annotation of protein-coding genes and other genomic components, and play significant roles in investigating potential interaction between TEs and functional genes (10).

Recently, several diploid and tetroploid *Gossypium* species genomes have been sequenced (11–15), and the availability of their draft genome sequences have provided an unprecedented opportunity for identification, structural and functional characterization and evolutionary analyses of TEs in this economically important crop. *Gossypium raimondii* (DD; 2n = 6), one of the putative D-genome parent of tetraploid cotton species [such as *G. hirsutum* (L). and *G. barbadense* (L.)] has a smaller genome size (∼737.8 Mb) (12). So, we carried out the characterization of almost all families of TEs in *G. raimondii* genome using comprehensive methods, and constructed the *G. raimondii* transposable elements database (GrTEdb) in this study. We implemented web-based sequence browsing, searching, downloading and blast tool to help users easily and effectively to annotate the TEs or TE fragments in genomic sequences from *G. raimondii* and other closely related *Gossypium* species. Thus, GrTEdb provide the first web-based friendly user interface database of TEs in *Gossypium* species, and will also facilitate genome evolution analyses within or across *Gossypium* species, evaluating the impact of TEs on their host genomes, and investigating the potential interaction between TEs and protein-coding genes.

## Construction and content of the database

The assembled sequence of the *G. raimondii* genome was downloaded from http://www.phytozome.com (11). A combination of structure-based and homology-based approaches was employed to identify different TEs in the *G. raimondii* genome. LTR-retrotransposons were characterized according to the methods previously described by Ma *et al.* (2006) (16): first, the LTR-retrotransposons were identified using the LTR_STRUC software; then CROSS_MATCH was used to detect elements missed by the program. The alignments were performed between *G. raimondii* genome and the flanking LTRs of these LTR-retrotransposons, which generated by the LTR_STRUC. Different perl scripts were written to facilitate the data mining and analyses. Other Non-LTR-retrotransposons and DNA transposons (such as *L1*, *Mutator*, *PIF-Harbinger*, *CACTA* and *Helitron*) were detected following the protocol provided by Holligan *et al.* (2006) (17): the alignment were performed between the conservative sequences of transposase in *Arabidopsis thaliana* and *G. raimondii* genomes using tblastn, and the TSD and TIR were detected using some perl scripts. The detailed manual inspection was conducted to confirm each predicted element and to define its structure and boundaries. In addition, TEs were classified into different superfamilies and families as previously described (2, 17). Only elements with clearly defined boundaries and insertion sites were deposited in the GrTEdb database.

Based the above approaches, 14 332 TEs were structurally annotated and clearly categorized in the *G. raimondii* genome, and these elements are classified into seven distinct superfamilies based on the order of protein-coding domains, structures and/or sequence similarity, including 2929 *Copia-like* elements, 10 368 *Gypsy-like* elements, 299 *L1*, 12 *Mutators*, 435 *PIF-Harbingers*, 275 *CACTAs* and 14 *Helitrons* (Table 1). Based on the 80-80-80 rule (2). TEs that were assigned as *Copia-* and *Gypsy-like* elements superfamilies were then categorized into 199 and 218 distinct families respectively because of their large number in *G. raimondii*.

**Table 1.** Summary of the identified TEs in *G. raimondii*

Class	Order	Superfamily	Copy numbers
Retrotransposons	LTR	*Copia*	2929
		*Gypsy*	10 368
	LINE	*L1*	299
DNA transposons	TIR	*Mutator*	12
		*PIF-Harbinger*	435
		*CACTA*	275
	Helitron	*Helitron*	14
Total			14 332

## User interface

**Figure 1.**
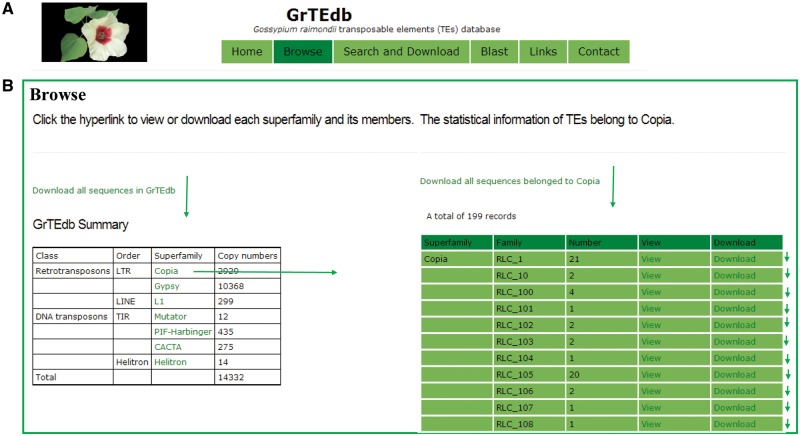
(**A**) The top menu of GrTEdb. (**B**) The user interface of browsing in GrTEdb. Users can browse the detailed information of each superfamily by clicking the hyperlinks provided in this page.

GrTEdb was established to enable users to browse, search, view, analyze and download the TEs data and information. The GrTEdb database organization is navigated by six sections: Home, Browse, Search and Download, Blast, Links and Contact (Figure 1A).

## Browse

In the browsing interface, the classification structures of TEs deposited in GrTEdb were showed. Users can download the whole TEs sequences, and can browse any one superfamily of interest by the hyperlinks provided. The detailed information of each superfamily can be retrieved and downloaded by clicking the corresponding entry (Figure 1B).

## Search and download

**Figure 2.**
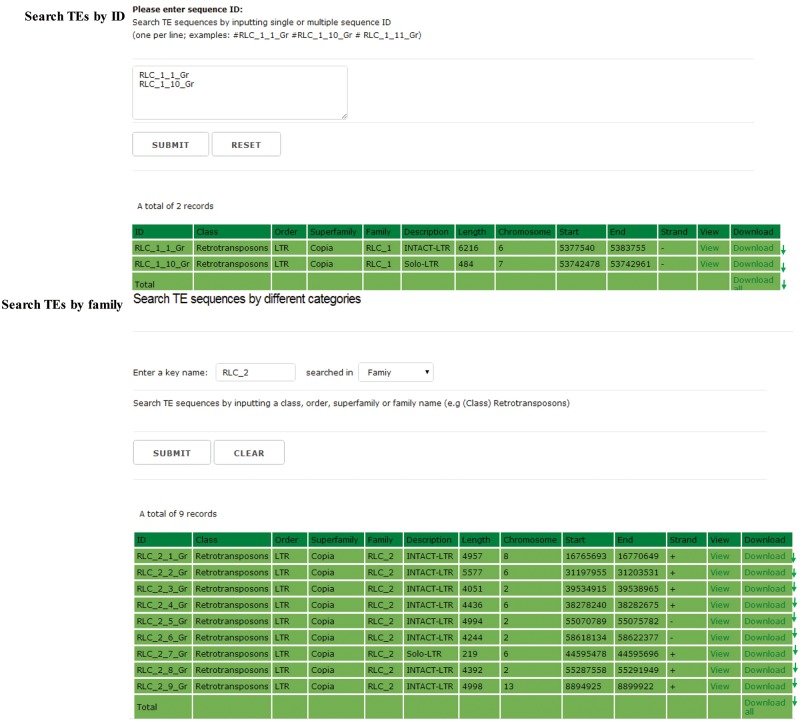
The searching interface of GrTEdb. Users can use a keyword to locate specific TEs quickly in GrTEdb (e.g. TE ID, Class, Order, Superfamily and Family). The search results can be viewed and downloaded by clicking the hyperlinks provided on the page.

In the searching and downloading interface, users can use a keyword to search the GrTEdb (e.g. TE ID, Class, Order, Superfamily and Family) to locate specific TEs quickly. The search results can be viewed and downloaded by clicking the hyperlinks provided on the page (Figure 2).

**Figure 3.**
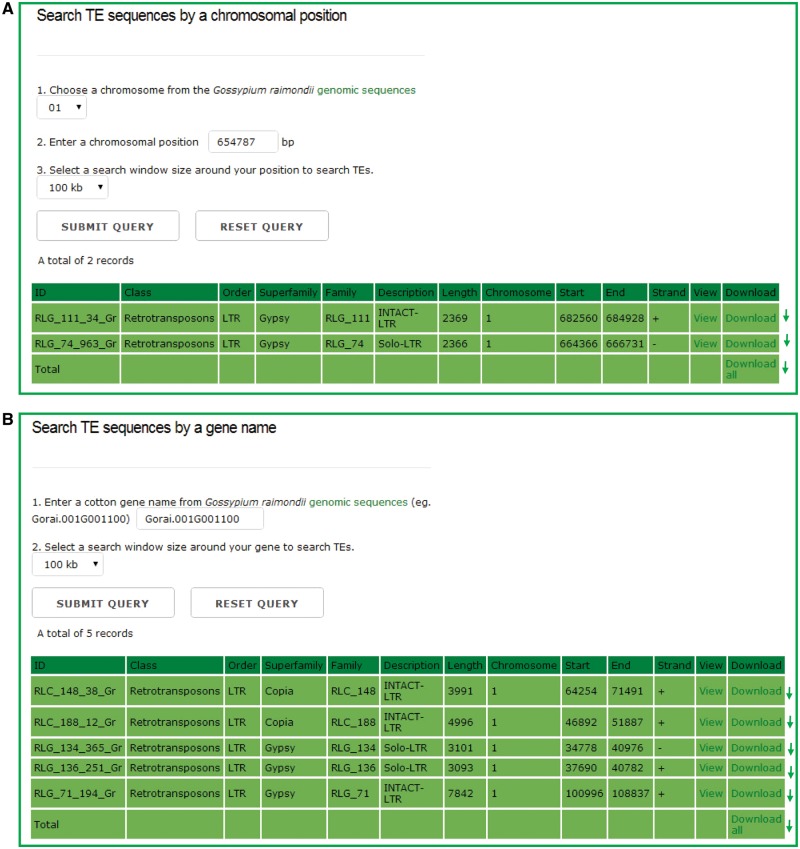
The chromosomal region search page. Users can retrieve the TE sequences for any one entire chromosome or in a defined window around either a chromosomal position or a gene model, and the detailed information of each retrieved TEs can be viewed and downloaded by clicking the hyperlinks provided on the page.

In the chromosomal region search page, users can retrieve the TEs for any one entire chromosome or in a defined window around either a chromosomal position or a gene model, and the detailed information of each retrieved TEs can be viewed and downloaded by clicking the hyperlinks provided on the page (Figure 3). This function can help users to locate TEs that surround the genes of interests easily, and study the interaction between TEs and their adjacent genes.

## Blast

**Figure 4.**
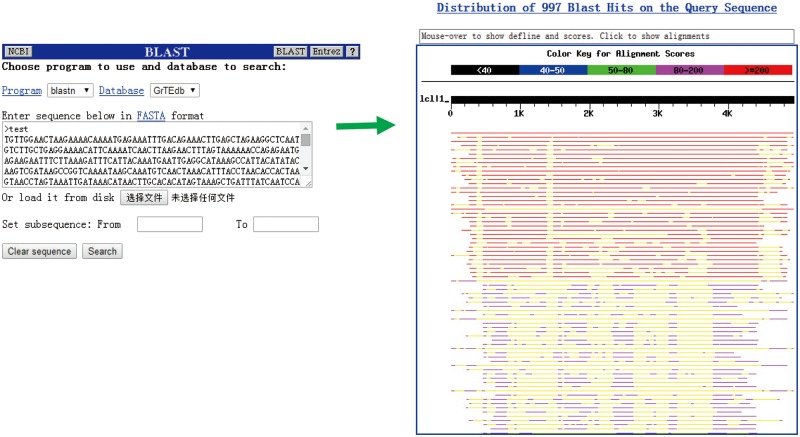
The BLAST interface (left) and a sample of BLASTn results (right) provided in GrTEdb.

We did not intend to integrate tools currently available (except for BLAST) for sequence comparison, editing and/or assembly in our database because of the complex structural variation and distribution patterns of TEs among classes and families (Figure 4). In the BLAST search page, users can handy and quickly compare their sequences with the cotton TEs deposited in GrTEdb.

## Links

A variety of links to other TEs database were included in our GrTEdb database.

## Contact

In this section, contact information and links to our labs were provided. Please feel free to contact us if you have any suggestions and problems.

## Discussion

Because of the structural complexity and the time consuming process, it remains challenging to annotate all TEs in a sequenced genome. Currently only a few TE databases have been established (10, 18–24). Because these databases can help users easily and quickly annotate their sequences, and they have been widely used (10). However, in these plant TE databases such as P-MITE (a Plant MITE database), the TIGR Plant Repeat Databases, and so on, there is little information about the cotton TEs. In parallel, although there were some reports associated with TEs in *Gossypium* (11–15, 25, 26), the web-based database of TEs was lacked. Here we have generated a web-based TE database (GrTEdb) using multiple methods, and only TEs with clearly defined boundaries were deposited in the database. More studies have showed that many TEs are structurally incomplete because they have undergone intra- or inter-element unequal recombination or accumulation of small deletions by illegitimate recombination (27, 28). For example, a large number of LTR-RT families with highly degraded protein-coding sequences or without any coding sequences (often defined as non-autonomous elements) have been found in several plants (29–35), and these elements remains challenging to be identified and characterized. Therefore, GrTEdb provides the reference sequences of TEs data for cotton, and users can use these data to identify more complex elements and develop their specific functions.

Recently, *G. arboretum* (A_2_) genome, a pupative contributor of the A subgenomes cotton species, and the allotetraploid upland cotton (AD)_1_ [*G. hirsutum* (L.)], which accounts for >90% of cultivated cotton worldwide, have been sequenced and assembled (13–15). Because of the close evolutionary relationships of DD, AA and AADD genomes, our GrTEdb database is not only useful for *G. raimondii* study, but also can facilitate structural and evolutionary analysis in AA, DD, AADD and other unfinished *Gossypium* genomes. The web-based interface can also help users at the beginning stage of bioinformatics to easily access and use this database. Further, TEs in our database will help cotton breeders develop markers for mapping agronomically important genes and accelerate breeding process.

## Conclusions

We have generated a web-based GrTEdb, and it provides researchers with not only resources and information related to different TEs in the cotton genome but also tools for performing data analysis. Thus, GrTEdb will facilitate cotton genome evolution analyses among AA, DD and AADD genome species, the evaluating impact of TEs on their host genomes, and investigating the potential interaction between TEs and protein-coding genes. In parallel, TEs in our database will facilitate users for marker development for mapping agronomically important genes, and for both intra- and inter-specific comparison of TEs at whole genome levels.

## Availability and requirements

All TEs or subsets of TEs can be viewed and downloaded from the website http://www.grtedb.org/, and all data deposited in the database are freely available to all users without any restrictions.

## Funding

The Key Scientific and Technological Project of Jiangsu Province (BK20150540); Jiangsu Agricultural Science and Technology Innovation Fund (CX(14)5008); the State Key Laboratory of Cotton Biology Open Fund (CB2016B03); the National Natural Science Foundation of China (NSFC) (31370266, 31471545).

*Conflict of interest*. None declared.
